# Mitochondria Dysfunction in Frontotemporal Dementia/Amyotrophic Lateral Sclerosis: Lessons From *Drosophila* Models

**DOI:** 10.3389/fnins.2021.786076

**Published:** 2021-11-24

**Authors:** Sharifah Anoar, Nathaniel S. Woodling, Teresa Niccoli

**Affiliations:** Department of Genetics, Evolution and Environment, Institute of Healthy Ageing, University College London, London, United Kingdom

**Keywords:** *Drosophila*, neurodegeneration, mitochondria, ALS, FTD

## Abstract

Frontotemporal dementia (FTD) and amyotrophic lateral sclerosis (ALS) are neurodegenerative disorders characterized by declining motor and cognitive functions. Even though these diseases present with distinct sets of symptoms, FTD and ALS are two extremes of the same disease spectrum, as they show considerable overlap in genetic, clinical and neuropathological features. Among these overlapping features, mitochondrial dysfunction is associated with both FTD and ALS. Recent studies have shown that cells derived from patients’ induced pluripotent stem cells (iPSC)s display mitochondrial abnormalities, and similar abnormalities have been observed in a number of animal disease models. *Drosophila* models have been widely used to study FTD and ALS because of their rapid generation time and extensive set of genetic tools. A wide array of fly models have been developed to elucidate the molecular mechanisms of toxicity for mutations associated with FTD/ALS. Fly models have been often instrumental in understanding the role of disease associated mutations in mitochondria biology. In this review, we discuss how mutations associated with FTD/ALS disrupt mitochondrial function, and we review how the use of *Drosophila* models has been pivotal to our current knowledge in this field.

## Introduction

Frontotemporal dementia (FTD) is the second most common early onset dementia, with Amyotrophic lateral sclerosis (ALS) representing the most prevalent motor neuron disease. While the two diseases are diagnosed based on distinct sets of symptoms, recent evidence suggests that they can in fact be considered two ends of the same disease spectrum, sharing genetic risk factors and neuropathological hallmarks (Ferrari et al., 2011; Van Blitterswijk et al., 2012). 15% of FTD patients also present with ALS, and 50% of ALS patients go on to develop FTD. In certain familial cases, different members of the same family will go on to develop either FTD or ALS (Martinaud et al., 2005). Pathologically, both diseases are characterized by intracellular proteinaceous inclusions, most commonly formed of the DNA/RNA binding protein TDP43, although aggregates can also be composed of superoxide dismutase 1 (SOD1), fused in sarcoma (FUS) or microtubule-associated protein tau (MAPT) (Ling et al., 2013; Turner et al., 2017), amongst others.

Amyotrophic lateral sclerosis, also called Lou Gehrig’s disease, is clinically characterized by the gradual loss of upper and lower motor neurons, eventually leading to death by respiratory failure. The average life expectancy is 2–5 years after diagnosis; however, 5–10% patients can survive up to 10 years after developing clinical symptoms (Millul et al., 2005; Mateen et al., 2010). There is no cure for ALS and the drugs that have been approved so far, such as Riluzole and Edavarone (Bensimon et al., 1994; Lacomblez et al., 1996; Pandya et al., 2013; Nguyen et al., 2018; Brown et al., 2020), only prolong life by 3–6 months (Nguyen et al., 2018). The overall global incidence of ALS is 1.59 in 100,000 people whilst the prevalence is 4.42 in 100,000 (Xu et al., 2020). The average age of onset is between 55 and 65 years of age, but for familial forms earlier ages of onset have been recorded (Orsini et al., 2015). Late-onset ALS is more prevalent in males than females, but the sex ratio is quite similar at younger ages (Manjaly et al., 2010). Sporadic ALS (sALS), in which no inherited mutation directly causes disease, accounts for 95% of cases. Conversely, familial ALS (fALS), accounting for the remaining 5% of cases, is associated with a number of genetic mutations which usually show a pattern of autosomal dominant inheritance (Byrne et al., 2011). More than 40 genes have been identified as carrying mutations causing ALS including angiogenin (ANG), matrin3 (MATR3) and vesicle-associated membrane protein (VAMP)-associated protein B (VAPB) (Alsultan et al., 2016; Gregory et al., 2020). Among these, the gene most commonly associated with pure ALS is SOD1, accounting for 20% of identified fALS cases (Orsini et al., 2015). Several other ALS-associated mutations lie in genes that have also been associated with FTD, including ataxin-2 (ATXN2), cyclin F (CCNF), Coiled-Coil-Helix-Coiled-Coil-Helix Domain Containing 10 (CHCHD10), chromosome 9 opening reading frame 72 (C9orf72), Dynactin (DCTN1), FUS, Optineurin (OPTN), TAR DNA binding protein (TARDBP or TDP-43), TANK-binding Kinase (TBK1), T cell-restricted intracellular antigen-1 (TIA1), Tubulin alpha 4A (TUBA4A) and valosin-containing protein (VCP) (Orr, 2011; Orsini et al., 2015; Greaves and Rohrer, 2019; Table 1). The intronic hexanucleotide repeat expansion (GGGGCC) in the C9orf72 gene (C9), is the most common genetic cause of both ALS and FTD, despite being identified only a decade ago (DeJesus-Hernandez et al., 2011; Renton et al., 2011).

**TABLE 1:** Major genes associated with ALS and FTD.

**Locus**	**Genes**	**ALS/FTD**	**Gene frequency**	**Mutations**	**Protein inclusions**	**Sources**
14q11.2	ANG	ALS	<1% ALS	Missense	TDP43	Greenway et al., 2006
5q31.2	MATR3	ALS	1% sALS 1-2% fALS	Missense	TDP43	Johnson et al., 2014; Nguyen et al., 2018
21q22.11	SOD1	ALS	5% sALS 20% fALS	Missense	SOD1/p62	Rosen et al., 1993; Shaw et al., 1998
20q13.32	VAPB	ALS	<1% ALS	Missense		Nishimura et al., 2004; Millecamps et al., 2010
12q24.12	ATXN2	ALS-FTD	4.7% ALS	CAG repeat	TDP-43	Elden et al., 2010; Bäumer et al., 2014; Rubino et al., 2019
16p13.3	CCNF	ALS-FTD	<1% sALS 0.6–3.3% fALS <4% FTD	Missense	TDP43	Williams et al., 2016; Nguyen et al., 2018
22q11.23	CHCHD10	ALS-FTD	<1% sALS 2% fALS <1% FTD	Missense/nonsense	TDP43	Bannwarth et al., 2014; Nguyen et al., 2018
9p21.2	C9orf72	ALS-FTD	6% sALS 37% fALS 5% sFTD 25% fFTD	G_4_C_2_ repeat expansion	TDP43/p62/dipeptides repeat/ubiquitin	DeJesus-Hernandez et al., 2011; Renton et al., 2011; Majounie et al., 2012
2p13.1	DCTN1	ALS-FTD	<1% sALS <1% fALS	Missense	TDP43	Puls et al., 2003; Münch et al., 2004, 2005
16p11.2	FUS	ALS-FTD	∼1% sALS 3% fALS <1% FTD	Missense/nonsense	FUS/p62	Kwiatkowski et al., 2009; Vance et al., 2009; Van Langenhove et al., 2012
10p13	OPTN	ALS-FTD	<1% ALS 4% FTD	Missense/deletion/nonsense	TDP43/p62	Maruyama et al., 2010; Van Langenhove et al., 2012; Pottier et al., 2015
5q35	SQSTM1/p62	ALS-FTD	2–3% ALS 2–3% FTD	Missense/nonsense/deletion	TDP43/p62	Fecto and Siddique, 2011; Elisa et al., 2012; Le Ber et al., 2013
12q14.2	TBK1	ALS-FTD	<1% sALS 3% fALS <1% FTD	Missense/nonsense	TDP43/p62	Cirulli et al., 2015; Freischmidt et al., 2015; Nguyen et al., 2018; Lamb et al., 2019
2p13.3	TIA1	ALS-FTD	<1% sALS 2.2% fALS <1% FTD	Missense	TDP43/p62	Mackenzie et al., 2017; Nguyen et al., 2018
2q35	TUBA4A	ALS-FTD	<1% sALS 1% fALS <1% FTD	Missense/nonsense	TDP43	Smith et al., 2014; Nguyen et al., 2018
9p13.3	VCP	ALS-FTD	1–2% fALS <1% FTD	Missense	TDP43/p62	Watts et al., 2004; Johnson et al., 2010
Xp11.21	UBQLN2	ALS-FTD	2% ALS <1% FTD	Missense	TDP43/p62/ubiquitin/FUS/OPTN	Deng et al., 2011; Synofzik et al., 2012
3p11.2	CHMP2B	FTD	<1% ALS <1% FTD	Missense/nonsense	Ubiquitin/P62	Skibinski et al., 2005; Parkinson et al., 2006; Van Langenhove et al., 2012
17q21.31	GRN	FTD	1–5% sFTD 5–20% fFTD	Missense/nonsense/splice site/frameshift	TDP-43	Baker et al., 2006; Cruts et al., 2006; Rademakers et al., 2012
17q21.2	MAPT	FTD	5–20% fFTD	Missense/splice site/deletion	Tau	Hutton et al., 1998; Rademakers et al., 2012

*Characteristics of major genes associated with ALS or FTD. Table is adapted from Hardy and Rogaeva (2014).*

Frontotemporal dementia, also called Frontotemporal Lobar Degeneration (FTLD) and formerly known as Pick’s disease, is a common early onset dementia (Ling et al., 2013). The prevalence is 15–22 per 100,000 individuals and the incidence is 2.7–4.1 per 100,000 individual per year (Onyike and Diehl-Schmid, 2013). The age of onset for FTD is usually below the age of 65 (Mercy et al., 2008; Seltman and Matthews, 2012). Clinically, FTD is characterized by atrophy of the frontal or temporal lobes and, depending on the initial presenting symptom, can been categorized as behavioral variant FTD (bv-FTD) if associated with changes of personality or behavior, or primary progressive aphasia (PPA) if associated with language impairment (Seltman and Matthews, 2012; Van Langenhove et al., 2012; Warren et al., 2013). There is no cure to date and no approved drugs to treat symptoms specific to this disease (Seltman and Matthews, 2012). The sporadic form of FTD accounts for 70% of cases, whilst familial forms accounts for 30% of the cases (Turner et al., 2017). The major genes linked with FTD are C9orf72, MAPT, progranulin (GRN) and chromatin modifying protein 2B (CHMP2B) (Turner et al., 2017; Casterton et al., 2020). Less common mutations have also been found in VCP, Sequestosome 1 (SQSTM1), OPTN and ubiquilin-2 (UBQLN2) (Table 1).

An increasing array of cellular processes have been linked to the development of both ALS and FTD (Mejzini et al., 2019; Liscic et al., 2020). One of the first mechanisms implicated in ALS development was excitotoxicity, caused by inappropriate elevation of intracellular sodium and calcium levels in response to sustained neuronal stimulation (reviewed in King et al., 2016). This disease mechanism is targeted by Riluzole, which blocks sodium currents and therefore reduces neuronal excitability. In the clinic, however, Riluzole only increases lifespan of ALS patients by a few months (Nguyen et al., 2018). More recent studies, fueled by the identification of a growing number of risk genes, have implicated other cellular pathways that could represent alternative therapeutic targets. For instance, many of the mutations associated with FTD/ALS fall in genes implicated in protein degradation and vesicle transport; impairments in these pathways could potentially underlie the observed accumulation of protein deposits in patients’ brains (for a review see Shahheydari et al., 2017). Similarly, impairments in RNA metabolism have been implicated by the RNA binding function of the disease-associated proteins TDP-43 and FUS. These proteins both translocate to the cytoplasm in disease, leading to disruptions in transcription and splicing (Lagier-Tourenne et al., 2010; Mackenzie et al., 2010). In addition, RNA metabolism dysfunction has also been linked to other disease-associated mutations such as C9 (Cooper-Knock et al., 2014). A number of other cellular processes are also disrupted: nucleocytoplasmic transport defects (Kim and Taylor, 2017; Fallini et al., 2020), impaired DNA repair (Sun et al., 2020), axonal transport defects, and cytoskeletal disorganization have all been described (Bilsland et al., 2010; Alami et al., 2014).

Increasingly, mitochondrial dysfunction has been implicated in disease progression: several FTD/ALS linked proteins (described in detail below) interact with mitochondria (Bannwarth et al., 2014; Tsai et al., 2020), and findings from both patient tissue and animal models link mitochondria dysfunction to FTD/ALS pathogenesis (Bannwarth et al., 2014; Nakaya and Maragkakis, 2018; Tsai et al., 2020). Moreover, mitochondrial impairments have been associated with other neurodegenerative diseases such as Alzheimer’s disease (AD) and Parkinson’s disease (PD) (Bosetti et al., 2002; Abou-Sleiman et al., 2006; Anandatheerthavarada and Devi, 2007; Cozzolino and Carrì, 2012), potentially suggesting some common pathogenicity across multiple disorders.

**FIGURE 1:**
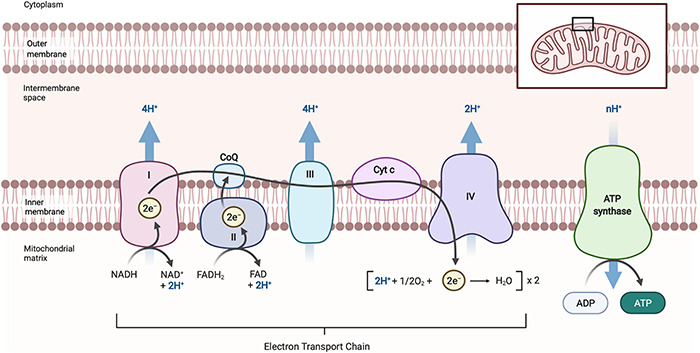
Schematic diagram of oxidative phosphorylation in mitochondria. Oxidative phosphorylation occurs at the inner mitochondrial membrane and is carried out by the electron transport chain, comprised of Complex I, II, III, IV, and ATP synthase. NADH (CI) and FADH (CII) donate electrons which then move along CoQ, CIII, cyt c and then CIV in a series of redox reaction that provide energy for the transfer of H^+^ ions across the intermembrane space to form an electrochemical gradient. Oxygen (O_2_), as the final electrons acceptor, is reduced to form water. The electrochemical gradient created in this process leads to H^+^ flowing back across the inner mitochondrial through ATP synthase, driving the phosphorylation of ADP to ATP. NAD, β-nicotinamide adenine dinucleotide; NADH, β-nicotinamide adenine dinucleotide 2-phosphate reduced form; FAD, flavin adenine dinucleotide; FADH, flavin adenine dinucleotide reduced from; CoQ, coenzyme Q (ubiquinone); CI, complex I; CII, complex II; CIII, complex III; CIV, complex IV; cyt c, cytochrome *c*; ADP, adenosine diphosphate; ATP, adenosine triphosphate. Images created with BioRender.com.

Neurons appear to be particularly susceptible to mitochondrial defects because of their high energetic demands (which almost exclusively have to be met by oxidative phosphorylation), their unique architecture with extremely long processes (axons can be longer than 1m in humans), and the fact they are extremely long-lived post-mitotic cells which cannot dilute out defective organelles by cell division. The high energetic demands of neurons are evident from the fact that the human brain contains about 2% of the body mass but requires about 20% of the oxygen consumption and energy needs of the whole body (Seager et al., 2020). As mitochondria are the main ATP producer within a cell (Figure 1), neurons have an abundance of mitochondria to meet their ATP requirements, which allow them to perform various cellular functions and activities (Kann and Kovács, 2007). If mitochondrial function is impaired, ATP production is not only diminished, but impaired oxidative phosphorylation (OXPHOS) can also generate inappropriately high concentrations of reactive oxygen species (ROS) that can further damage mitochondria (Bhatti et al., 2017) and eventually lead to neuronal death.

To investigate the roles of mitochondrial dysfunction and other disease mechanisms, both patient-derived cells and animal models have been essential scientific tools (Vandamme, 2014; Roberts and Speirs, 2017; Kim et al., 2020). With the advent of GWAS studies a multitude of mutations have been linked to disease development (Witte, 2010; Tam et al., 2019). Confirming that these mutations cause disease, and understanding how they lead to neurodegeneration, has often required the generation of animal models carrying these mutations. These models have allowed the field to build an initial understanding of how disease-associated mutations lead to declining organismal health generally and neurodegeneration specifically. Among animal models, the fruit fly *Drosophila* has proven invaluable in modeling neurodegenerative disease. Over 20 years ago, the first fly models of neurodegeneration showed, crucially, that over-expression of human disease genes in flies could not only lead to neurodegeneration (Warrick et al., 1998) but also mimic the main characteristics of the disease (Feany and Bender, 2000). Since then, a host of neurodegenerative disease models have been developed in flies, which have provided critical insights into disease pathology.

**FIGURE 2:**
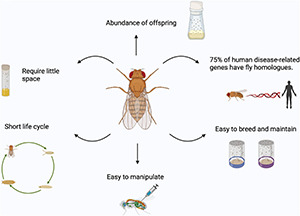
Advantages of using *Drosophila* as a model organism. Fruit flies are easy to breed and maintain in large numbers, easy to manipulate, require little space, have short life cycle with abundant offspring and about 75% of human disease-related genes have fly homologs. Images created with BioRender.com.

The success of flies stems from the fact that flies require little space, have short life cycles with abundant offspring, and are easy and inexpensive to breed and maintain in large numbers (providing strong statistical power) (Ashburner and Bergman, 2005; Figure 2). In this review, we will give a general overview of *Drosophila* as a model system to study FTD/ALS, with a focus on how *Drosophila* has been a powerful tool to study mitochondrial dysfunction in models of these diseases.

## *Drosophila* as a Model System

### *Drosophila* Biology

Since Thomas H. Morgan used *Drosophila* in his ‘Fly Room’ at Columbia University to study genetic inheritance in 1910, *Drosophila* has been widely used as a model organism. Thomas H. Morgan won the Nobel Prize in Physiology or Medicine in 1933 for formulating the chromosomal theory of inheritance, based on his findings looking at patterns of inheritance of the white eye pigment in flies. To date, 6 Nobel Prizes in Physiology or Medicine have been awarded to 10 researchers using flies in their research including Thomas’ proteges Allan Sturtevant, Calvin Bridges and Hermann Muller (Zhang K. et al., 2018). These trailblazing studies underscore the fundamental contribution of this unassuming model organism to our current understanding of many aspects of biology.

*Drosophila* development and physiology are surprisingly similar to those in more complex eukaryotes. For example, *Drosophila* has a similar dorsoventral patterning to vertebrates with the ventral area of *Drosophila* being analogous to the dorsal area in vertebrates (De Robertis and Sasai, 1996). Moreover, homologous genes control the patterning of the organism. Among these, decapentaplegic (*dpp*) is expressed in the dorsal region of *Drosophila*, whereas the vertebrate homolog, bone morphogenetic protein 4 (*bmp-4*), is expressed in the ventral region. Similarly, short gastrulation (*sog*) in *Drosophila* is expressed in the ventral region, whilst it vertebrate homolog, chordin (*chordin*), is expressed in the dorsal region (Holley et al., 1995). *Drosophila* also shows strong similarity to vertebrates in organ development: Hedgehog (Hh) in *Drosophila*, has a similar limb patterning function as Sonic hedgehog (SHH) in vertebrates (Riddle et al., 1993), and Paired Box 6 (*Pax6)* in both flies and vertebrates has analogous functions in eye development (Wawersik and Maas, 2000). Finally, the components of major signaling pathways, such as Wingless and Notch, are also conserved between flies and vertebrates (Baker, 1987; Fleming, 1998; Bejsovec, 2018). The conservation extends to genes and signaling pathways modulating longevity and aging, a field in which *Drosophila* has provided fundamental insights (Piper and Partridge, 2018). For instance, the discovery that *chico* (insulin-receptor substrate) and *InR* (insulin-like receptor) mutants extended lifespan in flies (Clancy et al., 2001; Tatar et al., 2001) prompted subsequent studies confirming similar findings in vertebrate models (Selman et al., 2008). Metabolic pathways are also highly conserved between mammals and *Drosophila*, making flies a valuable model system for metabolism research such as in diabetes and obesity (Baker and Thummel, 2007; Bharucha, 2009).

Flies are also the simplest model organism extensively used in research with a complex body pattern and many organs are analogous to those in humans. For example, flies have a gut, a beating heart, and a clearly defined central and peripheral nervous systems, allowing them to model multisystem phenotypes associated with disease mutations. These features have enabled *Drosophila* to provide numerous insights in the study of complex human diseases such as cancer, cardiovascular diseases, and neurodegenerative diseases. For instance, *Drosophila* has been used to identify and screen drugs for cancer therapeutics (Hirth, 2010; Bangi et al., 2019), and to study embryonic cardiac development and adult cardiac performance (Piazza and Wessells, 2011).

*Drosophila* has also been used extensively to study neurodegenerative diseases. Although the *Drosophila* brain is not as anatomically complex as the mammalian one, the cellular and molecular characteristics of the nervous system are broadly similar. At the cellular level, *Drosophila* and mammalian nervous systems both possess functionally distinct categories of neuronal and glial cells, and both possess a blood brain barrier (Stork et al., 2012; Kasture et al., 2018). Flies also display a range of complex behaviors such as sleep, learning and memory, courtship, and navigation. Importantly, these behaviors decline with age and in the presence of toxic genes associated with disease in humans, making flies a useful tool to study neurodegenerative diseases (Buchanan and Benzer, 1993; Jackson et al., 1998; Warrick et al., 1998).

At the genome level, *Drosophila* is simpler than vertebrate models, with only 4 chromosomes and 13,821 genes. However, the completed sequencing of the *Drosophila* genome has shown that about three-quarter of human disease-related genes have homologs in *Drosophila* (Reiter et al., 2001). Moreover, the *Drosophila* genome has relatively low genetic redundancy: classes of genes with multiple members in humans are often represented by only a single orthologous gene in flies. *Drosophila* research also benefits from its own comprehensive, easily searchable, database, known as FlyBase, where all published information about flies is cataloged soon after publication. This impressive repository provides researchers with access to a wide range of information, allowing them to coordinate their studies more efficiently. Because *Drosophila* as a model organism has been studied for over 100 years, the field has also developed a vast array of tools for manipulating and monitoring gene expression, placing *Drosophila* at the forefront of large-scale genetics screens. Classical X-ray radiation exposure, feeding with the mutagen ethyl methane sulfonate (EMS) (Greenspan, 1997), and the development of transposable P-element insertions have helped produce massive mutant strain collections which can be used to investigate different biological processes. Large collections of *Drosophila* strains have also been generated for over-expression of genes of interest (EP lines and FlyORF lines), as well as for RNAi-mediated down regulation of endogenous genes (Trip and VDRC RNAi lines) (Bellen et al., 2004; Dietzl et al., 2007; Bischof et al., 2013; Perkins et al., 2015) (Additional information about *Drosophila* stocks, reagents, online tools and key resources are reviewed extensively in Mohr et al., 2014). The presence of these large collections dramatically speeds up research by saving individual researchers the time and effort of designing and developing new lines. Moreover, the cell-type-specific effects of gene overexpression or knockdown can be studied using bipartite expression systems such as GAL4/UAS (upstream activating sequence) (Brand and Perrimon, 1993).

**FIGURE 3:**
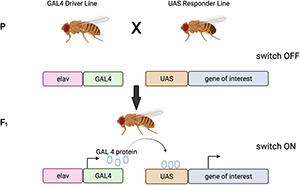
GAL4/UAS bipartite system of expression. The driver line contains a tissue specific promoter (in this case the neuronal specific elav promoter), driving the expression of yeast GAL4 protein in neurons. The responder line carries the gene of interest cloned down-stream of the UAS promoter, this, however, is not expressed. When the driver line is crossed to the responder line, the GAL4 driver and the UAS-transgene construct will come together in one animal. The GAL4 will be expressed in neurons, bind the UAS promoter and drive the expression of the gene of interest specifically in neurons. Images created with BioRender.com.

The GAL4/UAS bipartite system is an extensively used tool for controlling gene expression in flies (Brand and Perrimon, 1993). It takes advantage of the yeast transcriptional activator GAL4, which is usually cloned downstream of a tissue specific enhancer (conferring a determined expression pattern). GAL4 binds to UAS, activating the transcription of the gene of interest cloned downstream of it, in a spatiotemporal manner matching the GAL4 expression pattern (Fischer et al., 1988; Brand and Perrimon, 1993). The fly line containing the GAL4 is often called the ‘driver,’ and the transgene fly line that carries the UAS is often called the ‘responder’ or ‘reporter.’ The two elements are kept as separate fly stocks so that the expression of the UAS line is only induced in the progeny of crosses between driver and responder lines (Figure 3). There are currently nearly 8000 GAL4 and derivative lines, offering an ever increasingly precise toolkit for cell-type-specific expression of transgenes^1^. Importantly, most of these lines are available from public repositories. In particular, there are a plethora of GAL4 lines for different brain cell types (Pfeiffer et al., 2008; Jenett et al., 2012), allowing studies of very specific neuronal or glial sub-populations (see Doherty et al., 2009; Luan et al., 2020).

Subsequent refinements to this system have also improved temporal control: the temperature-sensitive GAL80 protein suppresses the GAL4 activity at lower temperatures (16°C) but is inactivated at higher temperatures (29°C), allowing genes to be turned on via a temperature shift (Lue et al., 1987; Duffy, 2002). More recently, Gal4-Geneswitch has been developed, where the GAL4 DNA binding domain has been fused to a progesterone inducible element which is activated upon feeding of the drug RU486 (Osterwalder et al., 2001; Nicholson et al., 2008). This system has been extensively used in neurodegenerative disease models to restrict expression of toxic constructs exclusively to adult neurons, a particularly useful feature for modeling diseases of adulthood as it avoids any confounding developmental effects (Sofola et al., 2010).

The ease of access to large libraries of stocks has enabled numerous large-scale enhancer and suppressor screens. This type of screen has been extensively used in neurodegenerative disease models to help identify downstream pathways of toxicity (Bilen and Bonini, 2007; Butzlaff et al., 2015; Zhang et al., 2015; Belfiori-Carrasco et al., 2017). The aim of these screens has been to identify factors that worsen (enhance) or ameliorate (suppress) the phenotype associated with a particular disease model. One of the most easily and commonly screened characteristics in *Drosophila* is the compound eye. *Drosophila* eyes are ideally suited as a genetic screening tool for diverse biological process, since about two-thirds of essential genes in *Drosophila* are involved in eye development (Thaker and Kankel, 1992). Furthermore, the complexity of the *Drosophila* eye, which encompasses 800 ommatidia, each in turn containing 8 photoreceptors, means that quantifiable features such as the number of ommatidia affected can precisely reflect the severity of the phenotype (Bate and Arias, 1993; St Johnston, 2002). At a macroscopic level, the size and roughness of the eye can also provide an immediate, scorable measure of cell death, which can be used as a rapid phenotypic readout. This serves as an ideal tool to conduct high throughput and unbiased screens (Lenz et al., 2013). Other screenable phenotypes linked to neurodegeneration include climbing ability, viability, and lifespan. Importantly, screens in flies have highlighted a number of pathways involved in disease development which have later been shown to be conserved in mammalian systems (reviewed in McGurk et al., 2015).

### *Drosophila* Mitochondrial Biology

Unsurprisingly given their central role in eukaryotic biology, *Drosophila* and human mitochondria show a very high degree of conservation in both genetic architecture and biochemical pathways. At the genomic level, *Drosophila* mitochondrial DNA (mtDNA) has similar components to human mtDNA, although the human mtDNA genome is 16,559 bp, 3 kb shorter than the Drosophila mtDNA genome at 19,517 bp (Lewis et al., 1995). Human mtDNA contains 13 polypeptides, 22 tRNAs and 2 rRNAs encoding the same components as *Drosophila* mtDNA, albeit with a slightly different genomic order (Garesse and Kaguni, 2005). The molecular functions typically associated with mitochondria, such as OXPHOS, mitochondria transport, and biogenesis are highly conserved, as are the nuclear-encoded genes required for mitochondrial function (Chen et al., 2019). *Drosophila* has therefore been an excellent model system to study mitochondrial function, with relevance to mitochondrial biology broadly and to neurodegeneration specifically.

One of the earliest examples of using *Drosophila* to study mitochondrial biology was the characterization of phenotypes in flies mutant for the *technical knockout* (*tko*) gene (Royden et al., 1987). *Tko* is the nuclear-encoded mitochondrial ribosomal protein S12 (mRpS12) (Mariottini et al., 1999). The *tko^25*t*^* mutant has reduced complex I, II, and IV activity and reduced ATP synthesis (Toivonen et al., 2001). This disruption in the electron transport chain (ETC) leads to a number of observable phenotypes, including seizure sensitivity, larval developmental delay, hearing impairments, and defective male courtship (Toivonen et al., 2001). Remarkably, these behavioral defects resemble the key features in patients with such disorders (Jacobs et al., 2004), providing early evidence that fly models could be used to model human mitochondrial disease. Indeed, several mitochondrial diseases models have since been developed which emulate the physiological symptoms seen in patients with mitochondrial disorders (Jacobs et al., 2004; Xu et al., 2008; Fernández-Ayala et al., 2010). Beyond mitochondrial diseases specifically, *Drosophila tko* mutants have been used in epilepsy research as the *tko* bang sensitive (bs) paralytics mutant displays similar features to those seen in human seizure disorders (Song and Tanouye, 2008; Whelan et al., 2010).

*Drosophila* models have also proved useful in defining the phenotypes most affected by defective mitochondria. For example, *Drosophila* studies defined mitochondria’s key role in sperm development and male fertility. Fuzzy onions (fzo), the founding member of the Mitofusin (Mfn) family of proteins, was first characterized in flies. Mutant *fzo1* mitochondria are unable to fuse in early spermatocytes, disrupting mitochondria organization and leading to male sterility (Hales and Fuller, 1997). Notably, these phenotypes have been replicated in mammalian models and observed in human patients (Zhang et al., 2016).

Finally, fly models have also proved invaluable to understanding the role of mitochondria in neurodegenerative disorders. For example, following the identification of mutations in *PINK1*, a PTEN-induced serine/threonine kinase 1, and in the E3 ubiquitin ligase Parkin, as causative genes in monogenic PD, flies were instrumental in identifying the role of these genes in mitochondrial biology (Clark et al., 2006; Park et al., 2006; Deng et al., 2008; Poole et al., 2008; Yang et al., 2008). Flies were used to show that both PINK and Parkin were implicated in the same biological pathway, playing a fundamental role in mitochondrial dynamics and degradation of dysfunctional mitochondria via mitophagy (Clark et al., 2006; Park et al., 2006; Deng et al., 2008; Poole et al., 2008; Yang et al., 2008).

### Assays for Studying Mitochondrial Biology

The study of mitochondria in flies is bolstered by a large number of assays to characterize mitochondrial form and function in detail, both in live and fixed tissue of wildtype and mutant *Drosophila* (Sen and Cox, 2017).

A number of general purpose dyes have been developed to visualize mitochondria; nearly all of these can be used in *Drosophila* tissues. The cell-permeant mitochondrion-selective dye MitoTracker Green has been used to observe mitochondrial mass (Rana et al., 2017). Specifically, MitoTracker Green labels the mitochondria matrix by covalently binding free thiol groups on cysteine residues belonging to mitochondrial proteins (Presley et al., 2003). A number of dyes have also been developed to measure mitochondrial membrane potential (MMP), a parameter that correlates with the mitochondrial ability to generate ATP. Tetramethylrhodamine, methyl ester (TMRM) or Tetramethylrhodamine, ethyl ester (TMRE), and 5,5′, 6,6′-tetrachloro-1,1′,3,3′-tetraethyl-benzimidazolylcarbocyanine iodide (JC1) dyes are some of the most commonly used dyes used for this purpose. TMRM, TMRE, and JC-1 are all fluorescent cationic dyes that accumulate in mitochondria based on their potential, the more polarized mitochondria are, the more the dyes accumulate. For TMRM and TMRE this results in a brighter signal at high MMP (Perry et al., 2011). JC1, on the other hand, changes color according to its concentration, forming red aggregates at high concentrations in healthy mitochondria, and remaining as a green monomer in mitochondria with low MMP (Sivandzade et al., 2019). The MMP in this case is measured by the ratio of the red/green fluorescence intensity. Finally, MitoSOX dye can be applied to measure ROS production (Yarosh et al., 2008). This fluorescent cationic dye is a derivative of dihydroethidum; in the presence of superoxide, MitoSOX is selectively oxidized in the mitochondrial matrix of live cells, where it emits a red color (Roelofs et al., 2015).

In addition to dyes, a host of fluorescent genetically encoded probes and bio-sensors have also been developed, particularly in *Drosophila*. As the genes encoding these sensors are usually under the control of the UAS promoter, they can be driven by cell-type specific GAL4 drivers. This allows monitoring of mitochondria morphology, physiology, and key metabolites *in vivo* in specific cell populations (Table 2).

**TABLE 2:** Genetically encoded mitochondrial biosensors/tools developed in *Drosophila*.

**Sensor line**	**Sensor type**	**Brief description**	**References**
UAS-AT1.03NL/UAS-AT1.03RK	ATP sensor	FRET sensor for live imaging, consisting of an ATP binding sequence located between two fluorophores. Upon ATP binding the two are brought in close proximity leading to increased FRET signal.	Tsuyama et al., 2013
UAS-mitoGFP UAS-mitoDSRed	Mitochondrial marker	Green or Red fluorescent protein tagged with a mitochondrial import sequence (acting as targeting signal to the mitochondrial matrix), used to visualize all mitochondria in live or fixed tissue.	Lutas et al., 2012; DeVorkin et al., 2014; Chowdhary et al., 2017; Morris et al., 2020
UAS- mito-GCaMP3	Ca2 + sensor	Circularly permutated EGFP M13/Calmodulin fusion protein, tagged with a mitochondrial import sequence. Upon Ca2 + binding the construct undergoes a conformational change, increasing fluorophore output, thus allowing to monitor Ca2 + concentration in live cells.	Lutas et al., 2012; Morris et al., 2020
UAS-PercevalHR	ATP/ADP sensor	Fluorescent biosensor composed of three tandem copies of an ATP-binding protein fused to a circularly permuted monomeric Venus. It has a dual excitation spectrum, with a peak at 405 nm, enhanced by ATP binding and at 488 nm, enhanced by ADP binding. The ratiometric signal reports the occupancy and therefore ATP/ADP ration in live cells.	Morris et al., 2020; Wong et al., 2021
UAS-SoNaR	NADH/NAD + sensor	Fluorescent biosensor composed of a fusion of circularly permuted yellow fluorescent protein and an NADH-binding domain. It binds to NAD and NADH with two specific conformation, associated with a different excitation spectrum. The ratiometric signal reports the occupancy and therefore NADH/NAD + ratio in live cells.	Bonnay et al., 2020; Morris et al., 2020
UAS-mito-roGFP2-Grx1/UAS-mito-roGFP2-Orp1	REDOX sensors	Mitochondrially targeted, redox sensitive GFPs, with a dithiol/disulfide switch on their surface leading to measurable ratiometric fluorescent change in response to redox changes in live mitochondria. These are fused to glutaredoxin (Grx), which increases specificity for glutathione or a microbial Orp1 H_2_O_2_ sensor, making it specific for H_2_O_2_.	Albrecht et al., 2011; Morris et al., 2020
UAS-Dendra2.mito	Photoconvertible mitochondrial tag	Mitochondrially localized photoconvertible protein that can be irreversibly photoconverted from a green fluorescent form to a red fluorescent form, by UV-violet or blue light. It allows to monitor behavior of photoconverted mitochondria over time.	Hwang et al., 2014; Bertolin et al., 2018
UAS-mito-PyronicSF	Pyruvate sensor	Mitochondrially targeted pyruvate FRET sensor composed of a circularly permuted GFP fused to a bacterial pyruvate sensitive transcription factor. Upon pyruvate binding a conformational change leads to increased FRET signal.	Arce-Molina et al., 2020

*Drosophila* has also been a useful model system for developing tools to assess specific molecular products of mitochondrial activity. For example, the MitoB probe, which uses a ratiometric mass spectrometry approach to measure H_2_O_2_ in the mitochondrial matrix, was developed using *Drosophila* (Cochemé et al., 2011). The probe contains a triphenylphosphonium (TPP) cation which is then conjugated to an arylboronic acid target in the mitochondria matrix; the final product is then measured by liquid chromatography tandem mass spectrometry.

Another important assay to study mitochondrial physiology is the measurement of oxygen consumption, which is directly linked to OXPHOS activity as the final step of the ETC involves the reduction of oxygen to water. One assay for measuring oxygen consumption is the high-resolution Oxygraph-2k, also called Oroboros, which measures oxygen consumption as well as additional parameters such as ROS production, mitochondrial membrane potential, ATP production, Ca^2+^, and pH^2^. Oroboros measures oxygen uptake and concentration after the injection of substrates for different ETC components, directly to samples immersed in a specific solution within a detection chamber. It requires a small amount of sample, with only 2 flies being sufficient for an assay (Costa et al., 2013). Advantages of this system include its flexibility to study each complex individually and its ability to perform serial measurements in the same sample, and its relatively low cost (Perry et al., 2013). Nonetheless, this system is relatively slow and low throughput.

The Seahorse XF Extracellular Flux Analyzer (Seahorse Bioscience Inc.) was introduced more recently to enable high throughput testing, albeit at extra cost. The instrument can measure the rate of mitochondrial OXPHOS (through determination of the oxygen consumption rate) and glycolysis through extracellular acidification rate (ECAR). The system achieves higher throughput by using fluorescent sensor cartridges on 24 or 96 cell-culture microplates^3^. In addition, the microplate-based assay has 4 ports to inject substrates and/or inhibitors to assess multiple conditions consecutively (reviewed in Horan et al., 2012).

Finally, a number of spectrophotometric assays have also been developed to study the activity of mitochondrial enzymes or the concentration of individual metabolites. These assays possess the advantage of being relatively cheap, rapid and reliable (Birch-Machin and Turnbull, 2001; Barrientos, 2002; Barrientos et al., 2009). They provide information on the catalytic activities of each respiratory chain complex or combination of complexes by following the production or depletion of particular substrates which absorb or transmit light at specific wavelengths. The results are then usually standardized against citrate synthase activity, frequently used as a standard in mitochondria (Frazier and Thorburn, 2012). The downsides of these assays are that few samples can be measured at one time, and they generally require a relatively large quantity of sample (Janssen and Boyle, 2019).

### Assays to Assess Health and Behavior in *Drosophila*

As described earlier, mitochondrial defects in flies have physiological implications on the whole organism that result in alterations in behavior and lifespan. These can provide useful organism-level phenotypic readouts of mitochondrial defects. Lifespans is one of the most commonly used readouts for late onset phenotypes in flies. Because of the sensitivity of lifespan as an assay, thorough protocols have been developed to standardize the many factors that can influence lifespan (Piper and Partridge, 2016). The healthy lifespan of flies can be evaluated across ‘normal’ aging, or lifespan can be assessed in the presence of mitochondrial stressors such as paraquat or rotenone to investigate the flies’ sensitivity to oxidative stress (Menzies et al., 2005; Meulener et al., 2005).

In addition to shortened lifespan, impairments in mitochondrial function can lead to more subtle neurological defects and neurodegeneration in flies, as well as in humans. A large number of assays have been developed to monitor neuronal impairments in flies. For instance, ‘bang-sensitivity’ measures the time for flies to recover from paralysis after being strongly shaken to the base of a tube. It mimics epilepsy and seizures associated with mitochondrial disease, features found for example in mitochondrial encephalomyopathy with lactic acidosis and stroke-like episodes (MELAS) (Jacobs et al., 2004; Celotto et al., 2006; Burman et al., 2014). In addition, locomotor dysfunction can be measured in flies via a climbing or negative geotaxis assay, which quantifies the negative geotactic reflex of flies. When gently tapped to the bottom of a chamber, flies naturally climb upwards against gravity and toward light. Flies are allowed to climb for defined time, and the height climbed is scored as an easy parameter that correlates with general locomotor function (Benzer, 1967; Inagaki et al., 2010). The climbing assay is widely used in *Drosophila* neurodegenerative disease research as it is a rapid readout of neuromuscular function, albeit a readout that lacks information on the exact tissues and circuits involved in any observed deficits.

Locomotion, sleep, and circadian rhythms can also be monitored with the widely used *Drosophila* activity monitoring (DAM) system (Pfeiffenberger et al., 2010). Flies are placed in sealed tubes with food within a climate and light-controlled incubator; monitors then use infrared beams to record each time a fly walks past a defined point within the tube (an event). Events are continuously scored for 3–4 days to monitor activity. To study sleep, a significant body of *Drosophila* sleep research has come to the consensus that ‘sleep’ can be defined in the DAM system as zero events for a sustained period of 5 min or more (Beckwith and French, 2019). The DAM system can therefore easily record movement and sleep. Circadian rhythmicity can also be assessed by transitioning the flies to constant darkness and observing how strongly and with what period circadian activity cycles persist. All of these parameters are influenced by neuronal health, in flies as well as humans, and can be used as sensitive measures for the effects of mitochondrial mutations on brain health.

## Evidence of Mitochondrial Dysfunction in Frontotemporal Dementia and Amyotrophic Lateral Sclerosis

The roles of mitochondria in FTD/ALS development have been underscored by the identification of disease mutations in the gene encoding the mitochondrial CHCHD10 protein. CHCHD10 localizes to the mitochondrial intermembrane space and is enriched at cristae junctions. Although the precise role of CHCHD10 is still unclear, muscle biopsies and fibroblasts from patients exhibit mitochondrial morphology defects, disorganization of cristae, and respiratory chain deficiencies such as abnormal complex V assembly (Bannwarth et al., 2014). CHCHD10 mutations also affect the stability of mitochondrial DNA in skeletal muscle (Bannwarth et al., 2014). Disease associated mutations can lead to impaired CHCHD10 mitochondrial import (Lehmer et al., 2018), and model systems suggest the R15L and S59L variants can display loss of function and dominant negative phenotypes, leading to mitochondria damage, accumulation of cytoplasmic TDP-43, and consequent synaptic dysfunction (Woo et al., 2017).

Other mutations linked to FTD/ALS have also been described to affect mitochondria in multiple ways, suggesting potentially a common pathogenic mechanism downstream of multiple causal mutations. We outline briefly below some of the major mitochondrial defects associated with FTD/ALS disease variants; for a more comprehensive discussion please see Smith et al. (2019).

### Mitochondrial Morphology/Dynamics

Mitochondria are a highly dynamic organelles that can change shape through fission/fusion events. When mitochondrial dynamics are disrupted, mitochondrial morphology and distribution are disrupted, which impinges directly on mitochondrial function. In ALS and in disease models, a number of studies have found aberrant mitochondrial morphology. For instance, post-mortem studies of tissue from sporadic ALS patients show swollen and mis-shapen mitochondria in the spinal cord (Sasaki and Iwata, 2007). A number of transgenic models with SOD1 mutations also display aberrant dynamics and morphology: for example, SOD1 G93A SH-SY5Y and NSC-34 cell lines show disrupted mitochondrial dynamics with the fission protein dynamin-related protein (Drp1) level increased and optic atrophy 1 (Opa1), a mitochondrial fusion protein, decreased (Ferri et al., 2010). Similarly in SOD1 G93A transgenic mouse spinal cord, Drp1 and mitochondrial Fission 1 (Fis 1) proteins are increased, while levels of the fusion proteins Mfn1 and Opa1 are reduced (Liu et al., 2013). This is also recapitulated in transgenic mice over-expressing mutant SOD1 which display mitochondrial vacuolar degeneration (Kong and Xu, 1998; Jaarsma et al., 2001; Sasaki et al., 2004).

Studies focused on TDP-43 have also identified roles for this disease-associated protein in mitochondrial biology. In primary mouse neurons, overexpression of either wildtype or mutant TDP-43 decreases mitochondrial length and density in neurites; knock-down of TDP-43 exerts the opposite effect (Wang et al., 2013). Similarly, in fibroblasts derived from patients with mutations in TDP43, the mitochondria appear fragmented (Onesto et al., 2016), and EM analyses of TDP43 patient samples reveal cristae morphological defects (Wang et al., 2019).

**FIGURE 4:**
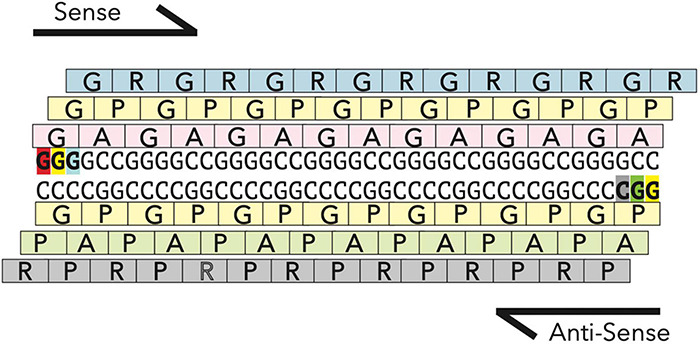
RAN translation in C9orf72 hexonucleotide repeat expansion. The hexonucleotide repeat expansion in the gene C9orf72 is transcribed both in the sense and antisense direction to generate two highly stable RNAs, which are translated via a non-canonical form of translation, called Repeat Associated non-ATG mediated (RAN) translation. RAN translation occurs in all frames along the transcript to generate five highly repetitive dipeptide repeat proteins (GR, GP, GA, AP, and PR). GR and PR being the most toxic (Mizielinska et al., 2014).

Hexanucleotide repeat expansions in the C9orf72 gene (Figure 4), the most common genetic cause of both FTD and ALS, also appear to cause mitochondrial deficits based on recent studies. Recent studies in a mouse model expressing one of the products of the C9 repeats, the dipeptide repeat protein GR, found age dependant inner mitochondria impairment and loss of cristae formation in the prefrontal cortex (Choi et al., 2019). Mitochondria in primary cortical neurons isolated from mice expressing 80 GR repeats, are also shorter in length and less motile compared to mitochondria in wildtype neurons. At the same time, the mitochondrial network in C9 patient derived fibroblasts did not appear substantially fragmented (Onesto et al., 2016). These differences are potentially due to the different cell types, as neurons may be particularly sensitive to mitochondrial dysfunction as discussed above. In addition to morphological differences, mitochondrial dynamics appear to be disturbed in some C9orf72 models. In a C9 mouse model expressing the dipeptide repeat GR, Drp1 levels are increased and Opa1 levels are decreased, similar to the findings from SOD1 mouse models (Choi et al., 2019). Additionally, induced pluripotent stem cells (iPSC)-derived motor neurons with a C9orf72 expansion show impairments in fast axonal transport of mitochondrial cargo (Mehta et al., 2021).

Fused in sarcoma, a gene in which mutations are associated with both FTLD and ALS, provides yet another example of a disease gene associated with mitochondrial deficits. EM analysis has shown morphological abnormalities in mitochondria from FUS patient spinal cord (Huang et al., 2010) and FTLD-FUS patient brain samples (Deng et al., 2015). In cultured neurons, expression of ALS-associated FUS mutants also leads to shortened mitochondria (Tradewell et al., 2012) and mitochondrial fragmentation (Deng et al., 2015, 2018).

### Calcium Homeostasis

Mitochondria play a central role in Ca^2+^ homeostasis as they store calcium for cell signaling activities and neurotransmitter release. Notably, disturbed mitochondrial calcium homeostasis may be a particularly early marker of pathogenesis in FTD/ALS. For instance, brain and spinal cord samples from mice expressing G93A or G85R mutant SOD1 demonstrate a reduction of calcium-loading capacity in mitochondria very early in the disease course, long before observable symptom onset (Damiano et al., 2006).

Key to the ability of mitochondria to manage Ca^2+^ levels is their interaction with the endoplasmic reticulum (ER), the other main Ca^2+^ store. ER-mitochondria contact sites (or MAMs) regulate Ca2 + exchange between the two organelles (Romero-Garcia and Prado-Garcia, 2019) and are instrumental in maintaining appropriate intracellular Ca^2+^ homeostasis. As with other mitochondrial phenotypes, MAMs are disrupted in several models of FTD/ALS associated mutations. Expression of either wildtype or mutant TDP-43 or FUS in human and mouse cells disrupts MAMs by disrupting the interaction of two key mediators of ER-mitochondrial interaction: resident ER protein; VAPB and the mitochondrial protein tyrosine phosphatase-interacting protein 51 (PTPIP51) (Stoica et al., 2014). C9orf72 hexanucleotide expansions also affect Ca^2+^ homeostasis. An extensive characterization of iPSC derived motor neurons from patients with C9orf72 expansions showed dysfunction in Ca^2+^ homeostasis, reduced levels of the antiapoptotic protein Bcl-2, increased ER stress, and reduced mitochondrial membrane potential, resulting in decreased cell survival (Dafinca et al., 2016). This suggests that a disruption of Ca^2+^ homeostasis is a common phenotype across multiple causal mutations.

### Reactive Oxygen Species

Excessive accumulation of ROS in cells can lead to cell damage and, eventually, cell death (Pizzino et al., 2017). Low levels of ROS perform essential signaling functions (Schieber and Chandel, 2014); however, excess ROS production leads to lipid peroxidation and oxidation of proteins and DNA. ROS are primarily generated in mitochondria as a by-product of oxygen and energy metabolism, making mitochondria a primary target of ROS damage. Excess ROS leads to nitration of mitochondrial proteins, mtDNA damage and mitochondrial respiratory chain dysfunction (Guo et al., 2013).

ALS and FTD, like many other neurodegenerative disorders, have been linked to excessive ROS production and consequent cellular damage. ROS has long been thought to be causally linked to ALS development as the first causal mutations linked to familial ALS were identified in SOD1, a superoxide scavenging enzyme localized to mitochondria whose primary role is to reduce ROS levels. ALS-associated mutations in SOD1 have been associated with increased ROS, with transgenic mice expressing G93A mutant human SOD1 showing oxidative damage to mitochondrial proteins and lipids (Mattiazzi et al., 2002; Vargas et al., 2011; Xiao et al., 2018; Chen et al., 2021; Méndez-López et al., 2021). In cell models as well, expression of G93A SOD1 leads to elevated ROS levels (Liu et al., 2002; Rizzardini et al., 2005) and increased lipid peroxidation level (Liu et al., 2002). Additionally, post mortem tissue from both carriers of SOD1 mutations and sporadic ALS patients display increased ROS-associated damage markers (Beal et al., 1997; Ferrante et al., 1997; Shibata et al., 2001).

Mutations in TDP43 and CHMP2B may also lead to excess ROS production by mitochondria. TDP-43 has been found to localize to mitochondria (Wang et al., 2016, 2019; Yu et al., 2020), and expression of mutant TDP-43 in fly neurons and mammalian cells lead to ROS over-production (Wang et al., 2019). Neurons with mis-localized TDP-43 showed increased ROS production relative to neurons with normal nuclear localization of TDP-43 (Wang et al., 2013). Neurons derived from FTD patients with CHMP2B mutations also show impaired cristae morphology, reduced mitochondrial respiration and increased accumulation of ROS relatively to healthy control neurons (Zhang et al., 2017).

C9 expansions can also lead to excessive ROS production in mitochondria. Motor neurons and fibroblasts derived from patients with expansions in C9orf72 show an age dependant increase in ROS production (Lopez-Gonzalez et al., 2016; Onesto et al., 2016). In fibroblasts, this is accompanied by an increase in oxygen consumption, ATP production and mitochondria hyperpolarization, possibly linked to an increase in mitochondrial biogenesis (Onesto et al., 2016). The mitochondrial dysfunction is potentially due to the highly positively charged GR, one of the repetitive dipeptides produced by the C9orf72 expansion. GR80 expressed in motor neurons has been observed to bind mitochondrial ribosomal proteins, leading to increased ROS production level and increased DNA damage; this is not the case with GA80, another C9-produced but uncharged repetitive protein (Lopez-Gonzalez et al., 2016). Reducing oxidative stress partially rescued DNA damage and partially suppressed GR80 induced cell toxicity in flies (Lopez-Gonzalez et al., 2016). Similarly, restoring the correct redox environment in SOD1 cellular models, also rescued cell death (Ferri et al., 2010), suggesting that ROS production is a driver of neurodegeneration for this model.

### Metabolism

Mitochondria are responsible for the majority of ATP production. When mitochondrial metabolism is disrupted, it affects the respiratory chain and ATP synthesis, which can be yet another mechanism of toxicity for mutations linked to FTD and ALS.

Studies from both patient tissue and disease models suggest that neuronal mitochondrial ATP production is reduced in FTD/ALS. In samples of ALS patients spinal cord, citrate synthase activity and respiration chain function are reduced (Wiedemann et al., 2002). In SOD1-G39A transgenic mice, the ETC is similarly disrupted, with mitochondrial respiration and ATP synthesis reduced at the time of symptom onset but not at earlier stages (Mattiazzi et al., 2002). C9orf72 expansions can also lead to reduced ATP generation: Complex I levels are reduced in C9 patients spinal cord and in C9 patient-derived iPSC cells, leading to a reduction in Complex I activity (Wang et al., 2021). Expression of poly(GR)80 in mice also leads to mitochondrial dysfunction by binding to the ATP5A1 (complex V), thus enhancing its ubiquitination and degradation (Choi et al., 2019). Moreover, studies using cultured neurons have shown mutant TDP43 binds and inhibits translation of mRNA of mitochondrially encoded Complex I subunits (Salvatori et al., 2018). Cells derived from patients carrying CHCHD10 mutations are also susceptible to energetic stress and display Complex I deficiency, leading to increased NADH/NAD + ratio and diminished tricarboxylic acid (TCA) cycle activity (Straub et al., 2021). Taken together, these studies show that mutations in several, different FTD/ALS-associated genes lead to similar metabolic energy deficiencies, again suggesting that specific mitochondrial impairments might be a common downstream pathogenic mechanisms.

## Fly Models of Frontotemporal Dementia and Amyotrophic Lateral Sclerosis and Their Role in Mitochondrial Dysfunction

Fly models of FTD/ALS generally use either gain-of -function (overexpression of the chosen gene) or loss-of-function (knockdown or knockout of the chosen gene) strategies to investigate disease pathogenesis. The overexpression and/or knockdown or knockout of the desired gene can exhibit a variety of phenotypes such as rough eye, climbing and crawling defects, eclosion defects, or wing defects, all of which suggest toxicity of the introduced genetic modification. These phenotypes can be used as quantifiable readouts of pathogenicity, helping researchers to investigate aspects of FTD/ALS pathogenesis with the goal of elucidating disease mechanisms. Similar to mammalian and human-derived cell models of disease, fly models of FTD/ALS generally display prominent mitochondrial defects, suggesting conserved mechanisms of toxicity and making flies a useful model system to investigate mitochondrial mechanisms of pathogenesis. We will focus here on examples from fly models of the most common disease risk factors: SOD1, TDP43, FUS, and C9orf72 hexanucleotide repeat expansions.

### Superoxide Dismutase 1 Fly Models

Superoxide dismutase 1 is the most extensively investigated gene associated with ALS, especially in rodent models. Of all the FTD/ALS risk genes, SOD1 is one of the most directly related to mitochondrial function as it localizes and aggregates at the intermembrane space (IMS) and the outer mitochondrial membrane (OMM) (Tafuri et al., 2015). SOD1 is a superoxide dismutase which binds zinc and iron and converts superoxide anions into oxygen and hydrogen peroxide (Pardo et al., 1995), thus playing a crucial role in reducing oxidative stress. To date, more than 200 variants have been found in the SOD1 gene^4^. The fly gene ortholog, also named SOD1, shows 67% amino acid similarity with the human sequence. A number of fly models have been generated looking at SOD1 function in disease development. Both gain and loss of function models have been generated, as it is still a matter of debate whether ALS caused by SOD1 mutations is due to a gain or loss of function of the protein (Mockett et al., 2003; Şahin et al., 2017; Agudelo et al., 2020).

Fly models expressing mutant SOD1 display motor defects (Bahadorani et al., 2013; Gallart-Palau et al., 2016), both when expressed in muscle (Gallart-Palau et al., 2016) and the brain (Bahadorani et al., 2013). In each case, locomotor dysfunction is accompanied by mitochondrial defects. Flies over-expressing zinc deficient human SOD1 also show cristae vacuolization and reduced ATP levels in fly heads but not in bodies (Bahadorani et al., 2013), and are severely sensitized to paraquat and zinc. Both paraquat and zinc act as mitochondria toxins, with paraquat inhibiting mitochondrial complex I activity and zinc interacting with nitric oxide to cause mitochondrial dysfunction. Flies expressing SOD1-G93A, an extensively modeled dominant causal mutation, also show defects in mitochondrial morphology (Gallart-Palau et al., 2016), similar to mitochondrial defects observed in mouse models of SOD1-G93A (Higgins et al., 2003). Taken together, these studies suggest the effects of SOD1 mutations on mitochondrial dysfunction are well conserved between flies and mammals.

### TDP-43 Fly Models

TDP-43 is an RNA/DNA binding protein involved in RNA metabolism. Neuronal inclusion bodies of hyperphosphorylated and ubiquitinated TDP-43 deposits were first described in the brain and spinal cord of FTD/ALS patients (Arai et al., 2006; Neumann et al., 2006). In healthy conditions, TDP43 localizes to the nucleus; however, in pathological conditions, TDP-43 translocates to the cytoplasm where it forms inclusions or aggregates (Ayala et al., 2008). The presence of cytoplasmic inclusions of TDP-43 suggests a toxic gain-of-function disease mechanism; in contrast, the loss of TDP-43 from the nucleus could also indicate a loss-of-function mechanism (Neumann et al., 2006; Winton et al., 2008; Giordana et al., 2010). TBPH is the fly TDP-43 ortholog, with 53% amino acid similarity between fly and human sequences. Reduction of TBPH, either by RNAi or genetic null mutants, reduces lifespan in flies and leads to a decline in motor function (Feiguin et al., 2009; Lu et al., 2009; Lin et al., 2011; Diaper et al., 2013), conversely, expression of human TDP-43 can rescue the loss of function phenotypes (Wang et al., 2011). Loss of TBPH function also causes reduced mitochondrial axonal transport linked to severe motor impairment (Baldwin et al., 2016), adding weight to TDP-43 loss of function as a disease mechanism. However, another study over-expressing wildtype human TDP-43 or A315T-mutant TDP-43 in the fly eye showed activation of mitochondrial unfolded protein response (UPRmt) and increased mitochondrial ROS levels in motor neurons (Wang et al., 2019), suggesting that both gain and loss-of-function of TDP43 can contribute to mitochondrial defects.

A number of studies have shown that over-expression of human wildtype TDP-43 in flies, either in muscle (Altanbyek et al., 2016) or neurons (Khalil et al., 2017), leads to aberrant mitochondrial morphology. Mitochondria appear fragmented, and the expression of well-known regulators of mitochondrial dynamics, such as mitochondrial assembly regulatory factor (Marf), which promotes fusion, are decreased both in muscle (Altanbyek et al., 2016) and brain (Khalil et al., 2017). Over-expression of Marf in muscle partially rescues mitochondrial morphology (Altanbyek et al., 2016), and its over-expression in neurons partially rescues the locomotor phenotypes and neuromuscular junction (NMJ) defects associated with TDP-43 over-expression (Khalil et al., 2017). Downregulation of a dynamin-like GTPase that mediates mitochondrial fission, Drp1, similarly leads to partial rescue of TDP-43 phenotypes in brain and muscle (Altanbyek et al., 2016; Khalil et al., 2017), confirming that dysfunction in mitochondrial dynamics contributes at least partially to TDP-43 toxicity. Similar defects to those in TDP-43 over-expression have been observed in flies over-expressing human FUS and TATA box-binding protein-associated factor 15 (TAF15), two additional ALS-associated genes, suggesting conserved mechanisms of pathogenicity (Altanbyek et al., 2016). The ease of generating transgenic flies and conducting rapid phenotypic analysis in flies allows for these comparisons across a number of models of disease, which would otherwise be more costly and time consuming in mammalian models of disease.

### Fused in Sarcoma Fly Models

Fused in sarcoma, similarly to TDP-43, is involved in RNA-related metabolism (Lagier-Tourenne et al., 2010) and is usually located in nucleus. Like TDP-43, abnormal cytoplasmic inclusions of FUS are formed in ALS. However, FUS and TDP-43 may act independently in ALS pathogenesis given the absence of TDP-43 pathology in FUS mutant patients (Kwiatkowski et al., 2009; Vance et al., 2009). Additionally, ALS patients with FUS mutations display a more aggressive disease form, with earlier symptom onset and shorter symptoms duration, as well as increased rate of bulbar onset (Yan et al., 2010). FUS is conserved in flies, where the orthologous gene *cabeza* (caz) shows 51% amino acid similarity to the human gene product (Stolow and Haynes, 1995). Importantly, expression of human FUS can rescue *caz* mutant phenotype (Wang et al., 2011), demonstrating functional equivalence of the orthologous genes.

As in TDP-43-mediated toxicity, FUS-mediated toxicity may result from either gain-of-function or loss-of-function mechanisms. In flies, overexpression of wildtype caz, over-expression of mutant caz P398L (carrying an equivalent mutation to the human pathogenic P525L mutation), or loss of caz can all result in inhibited mitochondrial transport, with mutant caz over-expression showing the most severe mitochondrial transport impairment and greatest effects on development and locomotor activity (Baldwin et al., 2016).

FUS fly models also display prominent mitochondrial defects: wildtype or P525L FUS over-expression in adult flies leads to mitochondrial fragmentation, as seen for TDP43 (Deng et al., 2015). Both wildtype and P525L mutant FUS interact with the catalytic subunit of mitochondrial ATP synthase (ATP5B) in cells and disrupt ATP synthase supercomplex (complex V) function and formation in flies, leading to the activation of the UPRmt (Deng et al., 2018). Down-regulation of ATP5B or genes involved in UPRmt partially rescues neurodegenerative phenotypes (Deng et al., 2018), suggesting that the impaired ETC and subsequent activation of the UPRmt can at least partially drive neurodegeneration. Downregulation of Hsp60, an ATP-dependent mitochondrial chaperone protein that modulates the localization of FUS to mitochondria in human cells, also partially rescues the rough eye phenotype and locomotor dysfunction observed in FUS transgenic flies (Deng et al., 2015).

These studies in flies suggest that the mitochondrial phenotypes associated with FUS and TDP43 fly models are very similar, pointing toward common downstream pathogenic mechanisms. Importantly, these findings suggest that therapies targeted to these mechanisms might be promising areas for study in multiple types of FTD/ALS.

### C9orf72 Hexonucleotide Repeat Expansion Fly Model

A hexanucleotide repeat expansion in the gene C9orf72 is the most common genetic cause of ALS and FTD (Chiò et al., 2012). Since this expansion is a relatively recently identified cause of FTD/ALS, much of our understanding of the pathogenic mechanisms of this expansion derives from models that can be rapidly generated and characterized – namely *Drosophila* models (Xu et al., 2013; Mizielinska et al., 2014; Freibaum et al., 2015).

Three mechanism of C9 toxicity have been proposed: (1) loss-of-function caused by reduction of endogenous C9orf72 transcription, (2) gain-of-function by formation of nuclear RNA foci transcribed from the G_4_C_2_ repeats, and (3) gain-of-function by the accumulation of highly repetitive dipeptide repeats proteins (DPRs) translated from the expanded repeats (Ash et al., 2013; Mori et al., 2013; Mizielinska et al., 2014). These DPRs can be translated from either sense (G_4_C_2_) or antisense (G_2_C_4_) transcripts through repeat-associated non-AUG (RAN) translation (Mori et al., 2013; Gendron et al., 2014). C9orf72 does not have a fly ortholog, so fly models have relied instead on overexpression of abnormal G_4_C_2_ repeats and DPR proteins to investigate potential gain-of-function mechanisms. The lack of an orthologous genes is both a strength and limitation of *Drosophila* in this case: the endogenous function of C9orf72 cannot be easily investigated using fly models, but the effects of the expansion can be isolated from the downregulation of the endogenous gene.

Over-expression of a variety of repeat lengths, from 30 G_4_C_2_ repeats to over 100, has been shown to lead to retinal degeneration in the eye, shortening of lifespan and impaired motor function (Xu et al., 2013; Mizielinska et al., 2014; Wen et al., 2014; Freibaum et al., 2015; Tran et al., 2015), suggesting that the repeats are highly toxic to neurons. As additional evidence of dose-dependent effects of toxicity, increasing repeat lengths show increased toxicity (Mizielinska et al., 2014). Mizielinska et al. (2014) showed that toxicity was most directly associated with the arginine-rich DPRs, GR and PR, a finding that has been confirmed in independent fly models (Wen et al., 2014; Tao et al., 2015; Yang et al., 2015; Boeynaems et al., 2016) as well as in mammalian systems (Shi et al., 2017; Zhang Y. J. et al., 2018; Choi et al., 2019; Zhang et al., 2019; Cook et al., 2020).

As seen in other genetic causes of FTD/ALS, G_4_C_2_ expansions can produce a number of mitochondrial deficits. Expression of G_4_C_2_(36) repeats disrupts mitochondrial transport when expressed in fly motor neurons (Baldwin et al., 2016). In fly muscle cells, poly(GR), which is highly positively charged, has been shown to enter mitochondria, disrupt cristae junction structure, increase membrane potential, and increase the formation of ROS (Li et al., 2020). GR80 binds to components of the Mitochondrial Contact Site and Cristae Organizing System (MICOS) complex, which is involved in cristae junction formation; the MICOS complex thus represents a potential mechanism by which GR80 can impair the exchange of mitochondrial metabolites and osmolytes. Consistent with this mechanism, downregulation of MICOS complex components in a fly model of GR toxicity rebalances ion homeostasis, reduces GR80 levels, and improves GR80 toxicity (Li et al., 2020). Rebalancing mitochondrial matrix ion levels with the K + /H + antiporter nigericin also rescues mitochondrial cristae morphology in GR(80) expressing flies (Li et al., 2020). Maintenance of appropriate MICOS complex activity is essential to mitochondrial homeostasis both in health and in disease: a recent study has found that knockdown of Drosophila MICOS genes phenocopies many of the features observed in C9orf72 models, including cristae junction loss in muscles, low mitochondrial membrane potential, imbalanced fusion/fission machinery, increased mitophagy, limited cell death, and reduced climbing ability (Wang et al., 2020). CHCHD10 mutations have also been implicated in MICOS complex integrity (Genin et al., 2018), again suggesting common mitochondrial related impairments driving toxicity in FTD/ALS. The role of MICOS might also not be limited to FTD/ALS: Pink1, a gene implicated in autosomal recessive Parkinson’s disease, has also been shown to regulate MICOS components (Tsai et al., 2018), suggesting common elements across multiple neurodegenerative disorders.

## Discussion/Conclusion

**FIGURE 5:**
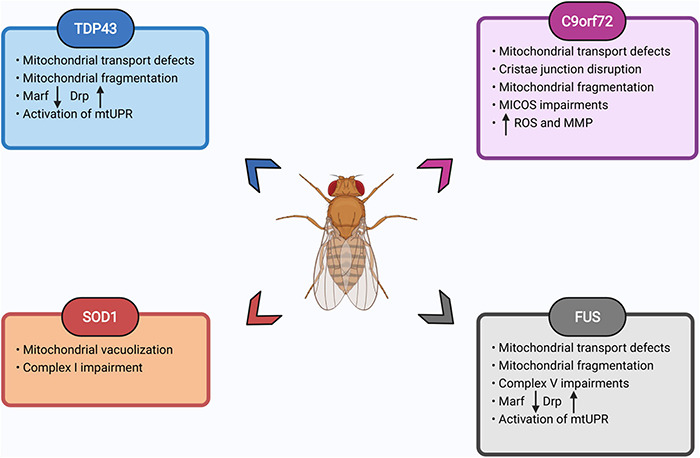
Summary of mitochondrial defects observed in fly FTD/ALS models. List of prominent mitochondrial impairments observed in fly models of TDP43, SOD1, FUS, and C9orf72 expansion. Please see text for details. Images created with BioRender.com.

In summary, *Drosophila* models of FTD/ALS display conserved pathological changes, demonstrating they can be rapid engines for the discovery of pathogenic mechanisms in neurodegenerative diseases. Flies have helped address questions about mitochondrial function and dysfunction in neurodegenerative diseases, aging, mitochondrial diseases, and developmental biology. Their extensive genetic tools, potential for large unbiased screens, and cost effectiveness has allowed *Drosophila* to provide key insights in understanding mitochondrial biology in FTD/ALS models (Figure 5). *Drosophila* is uniquely placed, given its facile genetics, to study convergent mechanisms of disease across multiple mutations, allowing genetic rescue experiments at scale to rapidly identify causative pathways in disease development. *Drosophila* also allows for screens to identify genetic or pharmacological interventions that ameliorate organismal health in addition to cellular toxicity, a potential advantage over cellular screening platforms.

In particular, flies could be used to address some of the following outstanding questions:

• In addition to GR, how does concurrent expression of all possible DPRs, and combinations of DPRs, from C9 repeats affect mitochondrial biology?
• Does the rescue of specific mitochondrial defects, for example MICOS integrity or Complex I activity, rescue lifespan and healthspan across models of multiple disease-associated mutations?
• Flies possess unique genetic toolsets that allow precise cell-type-specific and temporal control of gene expression. This places them in an ideal setting to address questions of cell-type and age-related susceptibility to disease. Why are certain neuronal populations most susceptible to the mutations that cause disease, and why only later in life? Are mitochondrial defects the first observable impairment, or do they follow other markers of neurodegeneration?

• Conversely, the same temporally controlled genetic tools can be used to answer questions related to therapeutic development. Can the rescue of a mitochondrial defect later on in disease development still rescue animal health, suggesting it could be a useful therapeutic avenue for patients already displaying symptoms?

Future research using *Drosophila*, particularly when complemented with confirmatory studies in mammalian or human systems, therefore has the potential to speed up and offer more thorough understanding of therapeutic strategies to address devastating neurodegenerative diseases.

## Author Contributions

SA and TN wrote the manuscript. NW edited the manuscript. All authors contributed to the article and approved the submitted version.

## Conflict of Interest

The authors declare that the research was conducted in the absence of any commercial or financial relationships that could be construed as a potential conflict of interest.

## Publisher’s Note

All claims expressed in this article are solely those of the authors and do not necessarily represent those of their affiliated organizations, or those of the publisher, the editors and the reviewers. Any product that may be evaluated in this article, or claim that may be made by its manufacturer, is not guaranteed or endorsed by the publisher.

## Footnotes

1
https://bdsc.indiana.edu/stocks/gal4/index.html

2
https://www.oroboros.at/index.php/o2k-concept/

3
https://www.agilent.com/en/products/cell-analysis/how-seahorse-xf-analyzers-work

4
https://alsod.ac.uk/output/gene.php/SOD1#variants
